# Overview of Calcitonin Gene-Related Peptide and Its Receptor

**DOI:** 10.1100/tsw.2001.401

**Published:** 2001-12-18

**Authors:** Nambi Aiyar

**Affiliations:** Cardiovascular Pharmacology, GlaxoSmithKline, King of Prussia, PA 19406, USA

## Abstract

Calcitonin Gene-Related Peptide (CGRP), a 37 amino acid peptide identified as the alternately spliced gene product of calcitonin gene, is a sensory neuropeptide with potent cardiovascular effects. CGRP is distributed throughout the central and peripheral nervous systems and possesses diverse biological actions. CGRP has been suggested to play a role in diseases such as migraine, diabetes, pain, and inflammation. Two forms of CGRP (alpha and beta) that differ in three amino acids have been identified and are encoded by different genes. Based on the differential biological activities of various CGRP analogs, the CGRP receptors have been classified into CGRP1 and CGRP2. Structure-activity studies of CGRP analogs showed that the C- and N-terminal regions of the peptide interact independently with their receptors. While C-terminal peptide, CGRP (8-37) behaves as a CGRP1 receptor antagonist, N-terminal peptide CGRP (1-12) behaves as a weak agonist. Structural modifications of CGRP(28-37) have yielded micromolar to nanomolar affinity ligands. CGRP receptor belongs to the calcitonin receptor like receptor (CRLR) family of G-protein-coupled receptors and has been shown to require a single transmembrane domain protein called receptor activity modifying protein-1 (RAMP1) for its functional expression as well as activity. Human, rat, and porcine CRLRs have been cloned and characterized. Currently, the major focus is on the identification of potent and specific nonpeptide antagonists for this receptor in order to understand the physiological and pathophysiological role of this peptide.

